# Accuracy of the Simplified Nutritional Appetite Questionnaire for Malnutrition and Sarcopenia Screening among Older Patients Requiring Rehabilitation

**DOI:** 10.3390/nu13082738

**Published:** 2021-08-10

**Authors:** Akio Shimizu, Ichiro Fujishima, Keisuke Maeda, Kenta Murotani, Tatsuro Inoue, Tomohisa Ohno, Akiko Nomoto, Junko Ueshima, Yuria Ishida, Ayano Nagano, Jun Kayashita, Naoharu Mori

**Affiliations:** 1 Department of Nutrition, Hamamatsu City Rehabilitation Hospital, Hamamatsu 433-8127, Japan; a.shimizu.diet@gmail.com; 2 Department of Palliative and Supportive Medicine, Graduate School of Medicine, Aichi Medical University, Nagakute 480-1195, Japan; j.ueshima@gmail.com (J.U.); okuda.yuria.785@mail.aichi-med-u.ac.jp (Y.I.); aya.k.nagano@gmail.com (A.N.); nmori@aichi-med-u.ac.jp (N.M.); 3 Department of Geriatric Medicine, Hospital, National Center for Geriatrics and Gerontology, Obu 474-8511, Japan; tatsuro-inoue@nuhw.ac.jp; 4 Department of Rehabilitation Medicine, Hamamatsu City Rehabilitation Hospital, Hamamatsu 433-8127, Japan; ifujishima@sis.seirei.or.jp; 5 Biostatistics Center, Kurume University, Kurume 830-0011, Japan; kmurotani@med.kurume-u.ac.jp; 6 Department of Physical Therapy, Niigata University of Health and Welfare, Niigata 950-3198, Japan; 7 Department of Dentistry, Hamamatsu City Rehabilitation Hospital, Hamamatsu 433-8127, Japan; tomohisa@zd5.so-net.ne.jp (T.O.); n.akiko-dent@sis.seirei.or.jp (A.N.); 8 Department of Clinical Nutrition and Food Service, NTT Medical Center Tokyo, Tokyo 141-8625, Japan; 9 Department of Nutrition, Aichi Medical University, Nagakute 480-1195, Japan; 10 Department of Nursing, Nishinomiya Kyoritsu Neurosurgical Hospital, Nishinomiya 663-8211, Japan; 11 Department of Health Sciences, Faculty of Human Culture and Science, Prefectural University of Hiroshima, Hiroshima 734-8558, Japan; kayashita@pu-hiroshima.ac.jp

**Keywords:** older adults, sarcopenia, malnutrition, poor appetite

## Abstract

This cross-sectional study aimed to examine the accuracy of the Simplified Nutritional Appetite Questionnaire (SNAQ) and the SNAQ for Japanese Elderly (SNAQ-JE) for the Global Leadership Initiative on Malnutrition (GLIM)-defined malnutrition and sarcopenia screening in older persons. We included 380 inpatients aged ≥65 years (mean age, 79.3 ± 7.9; 60.0% women) and admitted to rehabilitation units. Undernutrition and sarcopenia were diagnosed based on GLIM criteria and the Asian Working Group for Sarcopenia, respectively, using bioimpedance analysis. Poor appetite was defined as an SNAQ score of <14 points and an SNAQ-JE score of ≤14 points. The sensitivity, specificity, and accuracy of these tools for detecting poor appetite for GLIM-defined malnutrition and sarcopenia were assessed. The rates of GLIM-defined malnutrition and sarcopenia were 56.8% and 59.2%, respectively. The number of patients with poor appetite was 94 (24.7%) for the SNAQ and 234 (61.6%) for the SNAQ-JE. The sensitivity and specificity of the SNAQ measured against GLIM-defined malnutrition were 32.9% and 73.1%, respectively, and against sarcopenia were 29.8% and 70.2%, respectively. The sensitivity and specificity of the SNAQ-JE measured against GLIM-defined malnutrition were 82.6% and 51.0%, respectively, and against sarcopenia were 86.0% and 53.7%, respectively. The SNAQ-JE showed fair accuracy for GLIM-defined malnutrition and sarcopenia in older patients admitted to rehabilitation units.

## 1. Introduction

Poor appetite in older adults is recognized as a part of geriatric syndromes [1]. Its causes are multifactorial and include age-related physiological changes, morbidity, and social factors [2]. It is associated with physical disability and frailty [3,4] and is thought to lead to adverse outcomes such as morbidity and mortality [5]. In particular, poor appetite may lead to inadequate dietary intake and is associated with undernutrition and sarcopenia [4,6,7]. Early diagnosis and treatment of poor appetite in older people may prevent undernutrition, sarcopenia, frailty, and adverse outcomes.

Many screening tools have been developed for the early detection of undernutrition and sarcopenia. The Mini Nutritional Assessment Tool Short Form (MNA-SF) [8] is a screening tool for undernutrition in older adults that is associated with satisfactory accuracy [9]. Sarcopenia screening tools include SARC-F and calf circumference [6,10,11]. These screening tools do not directly assess poor appetite, although they may assess food intake, physical function, and muscle mass [1]. Since poor appetite is associated with undernutrition and sarcopenia, [3,4] screening for poor appetite may improve undernutrition and sarcopenia treatment.

The Simplified Nutritional Appetite Questionnaire (SNAQ) was developed to identify poor appetite in older people [12]. In addition, the SNAQ for Japanese Elderly (SNAQ-JE) has been validated for use in the Japanese older adult population [13]. The SNAQ has been shown to have sensitivity and specificity of approximately 70% each for predicting undernutrition in older inpatients [14,15,16,17]. However, previous studies have not validated the accuracy of the SNAQ against the Global Leadership Initiative on Malnutrition (GLIM) criteria, used to diagnose undernutrition in various regions and ethnicities [7]. The GLIM criteria are highly sensitive in diagnosing undernutrition in older inpatients and have good predictive validity for poor prognosis [18,19,20,21]. Therefore, determining the sensitivity and specificity of the SNAQ to the GLIM criteria may provide opportunities for interventions for both poor appetite and undernutrition. Similarly, sarcopenia is associated with worse functional outcomes, such as swallowing function, cognitive function, and activities of daily living (ADLs), in older inpatients [6,22]. Tools that help increase the rate of accurate sarcopenia diagnosis are required in clinical practice.

To the best of our knowledge, no previous study has examined the screening accuracy of the SNAQ and SNAQ-JE for GLIM-defined malnutrition and sarcopenia in older inpatients. Tools to assess poor appetite, including the SNAQ and SNAQ-JE, can predict future weight loss [12,13]. Thus, they are expected to play multiple roles, not only for screening current undernutrition and sarcopenia but also for screening patients who may develop or experience worsening undernutrition and sarcopenia in the future. In addition, the SNAQ-JE was developed for better adaptability in the Japanese older adult population [13]. Nevertheless, the SNAQ has been used in some studies of the Japanese older adult population [23]. Hence, we thought it necessary to compare the respective screening accuracies of the SNAQ and SNAQ-JE. Therefore, this study aimed to verify the screening accuracy of the SNAQ and SNAQ-JE for GLIM-defined malnutrition and sarcopenia in older patients.

## 2. Materials and Methods

### 2.1. Participants

This cross-sectional study was performed at the Hamamatsu City Rehabilitation Hospital (Shizuoka, Japan). This hospital provides rehabilitation services based on the Japanese insurance system. The participants were older hospitalized patients with stroke, musculoskeletal disease, or hospital-associated deconditioning, aged ≥65 years, and admitted to our rehabilitation units between January 2019 and January 2021. Patients who were able to answer the appetite questionnaire were included. Patients were excluded if they required non-oral intake, they could not answer the appetite questionnaire, they had pacemaker insertions, their bio-electrical impedance analysis (BIA) could not be measured, or their data collection was incomplete. This study was approved by the Ethics Committee of the Hamamatsu City Rehabilitation Hospital (ID: 20-63). Informed consent was not obtained because of the retrospective study design. Patients could withdraw from the study at any time, using the opt-out notification function available on the study website.

### 2.2. Measurements

Data on the characteristics of patients were obtained from their medical records, including age, sex, primary diseases, number of days from disease onset to admission in a rehabilitation hospital, height, weight, body mass index (BMI), comorbidities, muscle mass, handgrip strength, swallowing function, cognitive function, and ADLs. The Charlson comorbidity index (CCI) [24], an ordinal scale with weighted scores, was used to evaluate comorbidities, considering diseases that impact prognoses, such as congestive heart failure and malignancy. The higher the score, the worse the prognosis [24]. Height and weight were measured by nurses, and the BMI was calculated by dividing the weight (kg) by the square of the height (m^2^). Muscle mass was measured by a dietitian, using the BIA method as recommended by the Asian Working Group for Sarcopenia (AWGS) 2019 [10]. The BIA method was performed while patients were in the supine position, before lunch, with the bladder emptied and with clothing on. The skeletal muscle mass index was calculated by dividing the appendicular skeletal muscle mass (kg), measured using the BIA method, by the square of the height (m^2^). Handgrip strength was measured using a hand dynamometer (MG-4800; CHARDER Electronic, Taichung, Taiwan). These parameters were measured with the participant sitting in a chair or bed with their elbows bent at 90°. Measurements were taken twice for the right and left hands, and the highest value was used for analysis. Swallowing function was assessed by speech-language therapists, using the Food Intake Level Scale (FILS) [25]. The FILS is an ordinal scale, scored from 1 to 10 points, focusing on daily consumption; higher scores indicate better swallowing function [25]. The ADLs were evaluated by physical or occupational therapists, using the Functional Independence Measure (FIM) [26]. The FIM consists of 18 items, with scores ranging on an ordinal scale from 18 to 126 points; a higher FIM score indicates higher ADLs independence [26]. Cognitive function was assessed by occupational therapists, using the Mini-Mental Examination State (MMSE) [27]. The MMSE score ranged from 0 to 30 points, with lower scores indicating lower cognitive function. This information was obtained within 3 days of admission.

### 2.3. Appetite Assessment Tools: SNAQ and SNAQ-JE

The SNAQ and SNAQ-JE, which have been validated in Japanese older populations, were used to assess appetite [12,13,28]. The SNAQ and SNAQ-JE comprise ordinal scales, consisting of four domains: appetite, feeling of fullness, taste of food, and the number of meals per day for the SNAQ [12,28] and appetite, feeling of fullness, taste of food, and “general mood” for the SNAQ-JE [13]. It has been reported that the questionnaire items used in the SNAQ-JE are more widely distributed and have higher factor loadings than those used in the SNAQ for older Japanese populations [13]. The score for each item on the SNAQ and SNAQ-JE ranges from 1 (very poor, very sad, etc.) to 5 (very good, very happy, etc.) [12,13,28]. The total score for the SNAQ and SNAQ-JE is in the same range of 4–20 points [12,13,28]. Previous studies have reported that the SNAQ-JE may be more suitable than the SNAQ for use with older Japanese adults [13]. The SNAQ is a simple screening tool with good predictive ability for weight loss [12,29]. A total score of <14 points for the SNAQ and ≤14 points for the SNAQ-JE is considered indicative of poor appetite [12,13,28].

### 2.4. Diagnosis of GLIM-Defined Malnutrition

The MNA-SF [8] was used for nutritional screening; this was followed by an assessment of undernutrition using the GLIM diagnostic criteria. The MNA-SF consists of six items and has a score range of 0–14 points, with scores of ≤11 points indicating a risk of undernutrition [8]. The diagnosis of undernutrition was based on the GLIM criteria [7]. The GLIM criteria consist of phenotypic and etiologic criteria and involve two steps: risk identification and undernutrition diagnosis. In this study, the MNA-SF [8] was used for nutritional screening, and patients with a score of ≤11 points were considered for the diagnostic process. GLIM-defined malnutrition was diagnosed in individuals who met at least one of the phenotypic and etiologic criteria. The phenotypic criteria involved weight loss (5–10% within 6 months or >10% beyond 6 months), low BMI (<18.5 kg/m^2^ for patients aged <70 years or <20 kg/m^2^ for those aged ≥70 years), and decreased muscle mass (skeletal muscle mass index (SMI) of <7.00 kg/m^2^ for males and <5.70 kg/m^2^ for females) [7]. The etiologic criteria involved decreased food intake or assimilation (vomiting and dysphagia) and inflammatory conditions/disease burden (acute or chronic disease) [7].

### 2.5. Diagnosis of Sarcopenia

Patients with decreased muscle mass and low muscle strength were diagnosed with sarcopenia, according to the diagnostic algorithm of the AWGS 2019 [10]. In this study, the walking speed and five times sit-to-stand, which are part of the AWGS 2019 diagnostic criteria, could not be performed in all patients due to their physical disabilities [30]. Therefore, sarcopenia was diagnosed by a combination of low muscle strength and decreased muscle mass. Muscle strength was assessed using handgrip strength, and handgrip strength values of <28.0 kg and <18.0 kg for men and women, respectively, were defined as low muscle strength. Muscle mass was assessed using SMI; SMI values of <7.00 kg/m^2^ and <5.70 kg/m^2^ for men and women, respectively, were defined as decreased muscle mass.

### 2.6. Data Analysis

Quantitative variables included non-parametric and parametric values and were expressed as median (interquartile range) or mean ± standard deviation. Categorical variables were expressed as the number of patients (%). Comparisons between groups were made using the Mann-Whitney U test for non-parametric variables, *t*-test for parametric variables, and 𝜒^2^ test for categorical variables. Multivariate logistic regression analysis was performed with GLIM-defined malnutrition as the dependent variable in Model 1 and sarcopenia as the dependent variable in Model 2 to determine whether poor appetite assessed by the SNAQ and SNAQ-JE was independently associated. As confounding factors affecting appetite, variables reported in previous studies [31], such as age, sex, primary disease, number of days from disease onset to rehabilitation, comorbidities, cognitive function, swallowing function, nutritional status, and ADLs, were entered. We calculated sensitivity, specificity, accuracy, positive predictive value, and negative predictive value to evaluate the ability of poor appetite to predict the diagnosis of GLIM-defined malnutrition and sarcopenia. Poor appetite was defined as an SNAQ score of <14 points and an SNAQ-JE score of ≤14 points, based on previous studies [12,13,28]. In addition, the accuracy for GLIM-defined malnutrition and sarcopenia was analyzed using undernutrition as assessed by an MNA-SF ≤7 as a reference tool. The rating of validity test statistics followed the recommended cut-off points for sensitivity and specificity: a ‘good’ rating represented a sensitivity and specificity of >80%; a ‘fair’ rating represented a sensitivity or specificity of <80% but both >50%; a ‘poor’ rating represented a sensitivity or specificity of <50% [32]. To further assess the ability of the SNAQ and SNAQ-JE to predict the diagnosis of GLIM-defined malnutrition and sarcopenia, the area under the curve (AUC) for the SNAQ and SNAQ-JE was determined, using the receiver operating characteristic curve against GLIM-defined malnutrition and sarcopenia. The AUC values of the SNAQ and SNAQ-JE for GLIM-defined malnutrition and sarcopenia were compared using the DeLong test. All statistical analyses were performed using IBM SPSS version 23.0 (IBM Japan, Tokyo, Japan); *p*-values of <0.05 were considered indicative of a statistically significant finding.

## 3. Results

In total, 598 patients were admitted to our rehabilitation units during the study period. Of these, 28 patients had non-oral intake, 163 patients could not answer the appetite questionnaire, 15 patients had missing data, and 12 patients had BIA that could not be measured. Finally, 380 patients (mean age, 79.3 ± 7.9; 60.0% women) met the eligibility criteria and were included in the analysis. In this study, 94 and 234 participants were classified as having poor appetite in the SNAQ and SNAQ-JE, respectively (Table 1).

The prevalence of GLIM-defined malnutrition and sarcopenia among participants was 56.8% and 59.2%, respectively. In the SNAQ-JE, both men and women with poor appetite had lower muscle mass (*p* = 0.021 and *p* = 0.002, respectively) and lower grip strength (*p* = 0.007 and *p* = 0.001, respectively) than their counterparts. Furthermore, patients who showed poor appetite in the SNAQ-JE also had lower MMSE and FIM scores (*p* = 0.003 and *p* = 0.001, respectively). The prevalence of GLIM-defined malnutrition and sarcopenia was higher among patients with poor appetite than the intact group, as assessed with both tools (Table 2).

Multivariate logistic regression analysis showed that poor appetite identified by the SNAQ and SNAQ-JE was an independent factor for the GLIM-defined malnutrition (odds ratio (OR) 3.004, 95% confidence interval (CI) 1.651–5.464, *p* < 0.001, OR 2.754, 95% CI 1.669–4.545, *p* < 0.001, respectively). Only poor appetite in the SNAQ-JE was an independent predictor of sarcopenia (OR 1.940, 95% CI 1.176–3.198, *p* = 0.009) (Table 3).

Table 4 shows the predictive ability of poor appetite and reference tools as predictors for GLIM-defined malnutrition and sarcopenia. Poor appetite in the SNAQ had low sensitivity (approximately 30%) but high specificity (approximately 80%) for GLIM-defined malnutrition and sarcopenia. Meanwhile, poor appetite in the SNAQ-JE had a sensitivity of approximately 70% and a specificity of approximately 50% for GLIM-defined malnutrition and sarcopenia. The SNAQ-JE tended to be more sensitive than the SNAQ. In addition, undernutrition, as assessed by the MNA-SF, showed a sensitivity and specificity of approximately 70% each for GLIM-defined malnutrition and sensitivity and specificity of approximately 60% each for sarcopenia.

**Figure 1:**
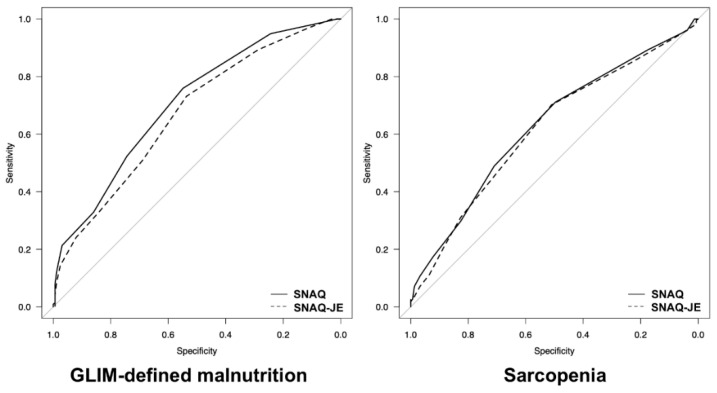
Comparison of the receiver-operating characteristic curves of the SNAQ and SNAQ-JE for GLIM-defined malnutrition and sarcopenia. Abbreviations: SNAQ: Simplified Nutritional Appetite Questionnaire; SNAQ-JE: Simplified Nutritional Appetite Questionnaire—Japanese elderly; GLIM: Global Leadership Initiative on Malnutrition.

The AUC associated with the SNAQ for GLIM-defined malnutrition was greater than that associated with the SNAQ-JE (0.706 vs. 0.627, *p* < 0.001 for DeLong test) (Figure 1). However, the AUC values of the SNAQ and SNAQ-JE for sarcopenia were comparable (0.670 vs. 0.616, *p* = 0.364 for DeLong test). Receiver operating characteristic curve analysis of the SNAQ and SNAQ-JE predictive value for GLIM-defined malnutrition and sarcopenia was performed. The AUC was 0.706 for the SNAQ and 0.627 for the SNAQ-JE for GLIM-defined malnutrition, and 0.670 for the SNAQ and 0.616 for the SNAQ-JE for sarcopenia. The AUC for GLIM-defined malnutrition was significantly higher in the SNAQ than in the SNAQ-JE (DeLong test *p* < 0.001). The AUC values of the SNAQ and SNAQ-JE for sarcopenia were comparable (DeLong test *p* = 0.364).

## 4. Discussion

This study investigated the screening accuracy of the SNAQ and SNAQ-JE for GLIM-defined malnutrition and sarcopenia in older patients who were admitted to our rehabilitation units. The results revealed fair accuracy of the SNAQ-JE in screening for GLIM-defined malnutrition and sarcopenia.

The SNAQ-JE showed fair accuracy for detecting GLIM-defined malnutrition. In this study, the accuracy of the SNAQ for detecting GLIM-defined malnutrition was associated with sensitivity and specificity estimates of 32.9% of 86%, respectively; meanwhile, the SNAQ-JE was associated with sensitivity and specificity estimates of 73.1% and 53.7%, respectively. The SNAQ is a validated tool for screening undernutrition in various settings [14,15,31]. The accuracy of SNAQ-based screening for undernutrition assessed by the MNA in inpatients has been shown to be poor to fair, with sensitivity and specificity estimates of 28–70.8% and 74.4–94%, respectively [14,15]. In contrast, the accuracy of nutritional screening tools for detecting GLIM-defined malnutrition in hospitalized patients has been shown to be fair to good, with sensitivity and specificity values of 47.1–84.1% and 47.1–93.7%, respectively [20,33,34]. These results suggest that the accuracy of nutritional screening tools for detecting undernutrition, as defined by the GLIM, is often better than fair. In addition, given the importance of early interventions in undernutrition [35], screening tools that show high sensitivity are desirable. In the DeLong test, the SNAQ had a greater AUC than the SNAQ-JE. However, the sensitivity of the SNAQ was lower than that of the SNAQ-JE. In addition, the MNA-SF, which was used as a reference tool in this study, showed better accuracy than the SNAQ and SNAQ-JE for undernutrition as defined by the GLIM, but the screening accuracy was fair. Therefore, the SNAQ-JE, which was rated with fair accuracy, as well as the MNA-SF may be useful in screening for GLIM-defined malnutrition in older Japanese inpatients. 

The SNAQ-JE showed fair accuracy for detecting sarcopenia. In this study, the accuracy of the SNAQ for detecting sarcopenia showed a sensitivity of 29.8% and specificity of 82.6%. In comparison, the SNAQ-JE showed a sensitivity of 70.1% and specificity of 51.0%. The diagnostic criteria for sarcopenia emphasize the need for screening, including SARC-F and calf circumference assessments, to enable early intervention [6,10]. A meta-analysis of studies investigating the diagnostic accuracy of the SARC-F for sarcopenia [36] in older adults reported sensitivity and specificity estimates of 21% (95% CI: 13–31%) and 90% (95% CI: 83–94%), respectively [37]. In addition, the calf circumference assessment recommended by AWGS 2019 [10] for screening sarcopenia in older inpatients is associated with a respective sensitivity and specificity of 85% and 66% in men and 91% and 28% in women [11]. In the present study, the SNAQ for sarcopenia was associated with sensitivity and specificity values of 29.8% and 82.6%, respectively; these values were similar to SARC-F values reported previously [37]. However, the SNAQ was not independently associated with sarcopenia in multivariate analysis. In addition, the MNA-SF, which was used as a reference tool in this study, showed better accuracy than the SNAQ and SNAQ-JE for sarcopenia, but the screening accuracy was fair. Therefore, the SNAQ-JE, which has fair accuracy for sarcopenia, may be a useful screening tool.

The SNAQ and SNAQ-JE may be useful for screening for GLIM-defined malnutrition and sarcopenia and for detecting prevention and intervention opportunities. The sensitivity and specificity values of predicting body weight loss of ≥5% within 6 months in patients with poor appetite identified by the SNAQ were 81.5% and 76.4%, respectively [29]. In addition, previous studies have shown that the SNAQ is associated with sensitivity and specificity values for predicting body weight loss of ≥10% of 88.2% and 83.5%, respectively [12]. Body weight loss is an essential component of malnutrition [7] and is considered a risk factor for sarcopenia [10]. The SNAQ is a simple, four-question instrument that does not require physical measurements and can help identify poor appetite in inpatients [12]. Therefore, the use of the SNAQ and SNAQ-JE in clinical practice may support early identification of poor appetite and the implementation of suitable interventions. In the current study, the distribution of “the number of meals per day” for the SNAQ was mostly concentrated in the response of “three meals per day”. This result was similar to the results of a previous study that created the SNAQ-JE [13] and SNAQ Turkish version [38]. In contrast, the distribution of responses to “general mood”, a component of the SNAQ-JE, was scattered. These results suggest that the low factor loading and narrow distribution of the “number of meals per day” factor of the SNAQ questionnaire shown in previous studies may be responsible for the differences in rates of poor appetite among older Japanese inpatients. Further studies are needed to investigate the predictive value of the SNAQ and SNAQ-JE in older Japanese inpatients, considering these findings.

This study has several limitations. First, the accuracy of the SNAQ and SNAQ-JE for GLIM-defined malnutrition and sarcopenia in this study was not very high. Therefore, prudence may be needed to determine the choice of nutritional therapy in individual clinical cases. Second, the study only included patients who were able to answer the SNAQ and SNAQ-JE. Therefore, the results of this study cannot be applied to all hospitalized patients. However, many comprehensive geriatric assessment tools have similar limitations [14]. Third, the accuracy of the SNAQ and SNAQ-JE may have been affected by the selective bias of the population included in this study. Approximately 60% of the participants in our study had GLIM-defined malnutrition and sarcopenia. In a previous study of Japanese patients undergoing rehabilitation, the prevalence of undernutrition and sarcopenia was approximately 40% and 50%, respectively [22]. Therefore, the accuracy of the SNAQ and SNAQ-JE for these conditions should be validated in the future in a multicenter study. Finally, the SNAQ-JE used in this study was a modified version of the SNAQ validated for older Japanese adults. Therefore, it is unclear whether similar results would be obtained with the SNAQ-JE for non-Japanese populations. However, the SNAQ has different cut-off values for different regions and settings [31]; therefore, it is necessary to use the cut-off value of the SNAQ that is appropriate for the target population.

## 5. Conclusions

The SNAQ-JE showed fair accuracy in screening for GLIM-defined malnutrition and sarcopenia in older patients admitted to rehabilitation units. Further studies are required to determine whether the SNAQ-JE can predict the development of GLIM-defined malnutrition and sarcopenia during hospitalization in older inpatients.

## Figures and Tables

**Table 1:** Patients’ epidemiological characteristics according to appetite status.

	SNAQ < 14	SNAQ ≥ 14	*p*-Value	SNAQ-JE ≤ 14	SNAQ-JE > 14	*p*-Value
Number of patients, *n* (%)	94 (24.7)	286 (75.3)		234 (61.6)	146 (38.4)	
Age, years ^1^	80.1 ± 8.1	79.1 ± 7.8	0.265	79.9 ± 8.0	78.5 ± 7.7	0.105
Sex (female), *n* (%) ^2^	64 (68.1)	164 (57.3)	0.070	143 (61.1)	85 (58.2)	0.592
Primary disease, *n* (%) ^2^						
- Stroke	36 (38.3)	136 (47.6)	0.011	106 (45.3)	66 (45.2)	0.365
- Musculoskeletal disease	47 (50.0)	140 (49.0)		112 (47.9)	75 (51.4)	
- Hospital-associated deconditioning	11 (11.7)	10 (3.5)		16 (6.8)	5 (3.4)	
Days from onset to rehabilitation ^3^	23 (15–34)	24 (17–34)	0.321	24 (17–34)	24 (17–33)	0.929
Charlson comorbidity index, points ^3^	1 (0–2)	1 (0–2)	0.662	1 (0–2)	1 (0–2)	0.473
Body mass index, kg/m^2 1^	20.6 ± 3.6	21.5 ± 4.3	0.065	20.9 ± 3.8	21.9 ± 4.52	0.012
SNAQ, points ^3^	12 (11–13)	15 (15–16)	<0.001	14 (13–15)	16 (16–17)	<0.001
SNAQ-JE, points ^3^	11 (10–12)	15 (14– 15)	<0.001	13 (11–14)	15 (15–16)	<0.001

^1^ *t*-test. ^2^ 𝜒^2^ test. ^3^ Mann–Whitney U test. Abbreviations: SNAQ: Simplified Nutritional Appetite Questionnaire; SNAQ-JE: Simplified Nutritional Appetite Questionnaire—Japanese elderly.

**Table 2:** Patients’ physical characteristics according to appetite status.

	SNAQ < 14	SNAQ ≥ 14	*p*-Value	SNAQ-JE ≤ 14	SNAQ-JE > 14	*p*-Value
Skeletal muscle mass index, kg/m^2 1^						
- Male	6.10 ± 1.09	6.61 ± 0.89	0.009	6.36 ± 0.95	6.72 ± 0.91	0.021
- Female	4.84 ± 1.04	5.13 ± 1.05	0.058	4.88 ± 1.05	5.33 ± 1.00	0.002
Handgrip strength, kg ^1^						
- Male	24.2 ± 7.8	26.8 ± 8.1	0.106	24.9 ± 7.7	28.4 ± 8.1	0.007
- Female	13.4 ± 5.7	15.3 ± 5.8	0.025	13.8 ± 5.4	16.3 ± 6.2	0.001
Mini-Mental Examination State, points ^2^	25 (21–28)	26 (21–29)	0.202	25 (20–28)	27 (22–29)	0.021
Food Intake Level Scale, points ^2^	8 (8–9)	8 (8–9)	0.414	8 (7–99)	8 (8– 9)	0.003
Functional Independence Measure, points ^2^	79 (65–94)	84 (66–99)	0.189	79 (61–95)	87 (72–102)	0.001
Malnourished (MNA-SF ≤ 7), *n* (%) ^3^	71 (75.5)	144 (50.3)	<0.001	150 (64.1)	65 (44.5)	<0.001
GLIM-defined malnutrition, *n* (%) ^3^	71 (75.5)	145 (50.7)	<0.001	158 (67.5)	58 (39.7)	<0.001
Sarcopenia, *n* (%) ^3^	67 (71.3)	158 (55.2)	0.008	158 (67.5)	67 (45.9)	<0.001

^1^ *t*-test. ^2^ Mann–Whitney U test. ^3^ 𝜒^2^ test. Abbreviations: SNAQ-JE: Simplified Nutritional Appetite Questionnaire; SNAQ: Simplified Nutritional Appetite Questionnaire—Japanese elderly; MNA-SF: Mini Nutritional Assessment—Short Form; GLIM: Global Leadership Initiative on Malnutrition.

**Table 3:** Results of logistic regression analysis for current GLIM-defined malnutrition and sarcopenia.

**Model 1: GLIM-Defined Malnutrition**			
	Odds ratio	95% confidence interval	*p*-value
Poor appetite in SNAQ	3.004	1.651–5.464	<0.001
Poor appetite in SNAQ-JE	2.754	1.669–4.545	<0.001
**Model 2: Sarcopenia**			
	Odds ratio	95% confidence interval	*p*-value
Poor appetite in SNAQ	1.627	0.894–2.963	0.111
Poor appetite in SNAQ-JE	1.940	1.176–3.198	0.009

Model 1 adjusted for age, sex, primary disease, days from disease onset to rehabilitation start, Charlson comorbidity index, Mini Mental State Examination, Food Intake Level Scale, and Functional Independence Measure. Model 2 adjusted for age, sex, primary disease, days from disease onset to rehabilitation start, Charlson comorbidity index, Mini Nutritional Assessment Short Form ≤7, Mini Mental State Examination, Food Intake Level Scale, and Functional Independence Measure. Abbreviations: SNAQ: Simplified Nutritional Appetite Questionnaire; SNAQ-JE: Simplified Nutritional Appetite Questionnaire—Japanese elderly; GLIM: Global Leadership Initiative on Malnutrition.

**Table 4:** Accuracy of poor appetite and reference tools as predictors of current GLIM-defined malnutrition and sarcopenia.

**Poor Appetite in SNAQ**						
	Sensitivity	Specificity	Accuracy	PPV	NPV	AUC
GLIM-defined malnutrition	0.329	0.860	0.558	0.755	0.493	0.706
Sarcopenia	0.298	0.826	0.513	0.713	0.513	0.627
**Poor Appetite in SNAQ-JE**						
	Sensitivity	Specificity	Accuracy	PPV	NPV	AUC
GLIM-defined malnutrition	0.731	0.537	0.647	0.675	0.603	0.670
Sarcopenia	0.702	0.510	0.624	0.675	0.541	0.616
**Undernutrition in MNA-SF ≤ 7**						
	Sensitivity	Specificity	Accuracy	PPV	NPV	AUC
GLIM-defined malnutrition	0.778	0.713	0.750	0.781	0.709	0.815
Sarcopenia	0.680	0.600	0.647	0.712	0.564	0.708

Abbreviations: SNAQ: Simplified Nutritional Appetite Questionnaire; PPV: positive predictive value; NPV: negative predictive value; AUC: area under curve; GLIM: Global Leadership Initiative on Malnutrition; SNAQ-JE: Simplified Nutritional Appetite Questionnaire—Japanese elderly; MNA-SF: Mini Nutritional Assessment—Short Form.

## Data Availability

Data sharing is not applicable to this article.
